# Dual-Frequency Soil Moisture Meter Method for Simultaneous Estimation of Soil Moisture and Conductivity

**DOI:** 10.3390/s24102969

**Published:** 2024-05-07

**Authors:** Jerzy S. Witkowski, Andrzej F. Grobelny

**Affiliations:** Faculty of Electronics, Photonics and Microsystems, Wroclaw University of Science and Technology, Wyb. Wyspiańskiego 27, 50-370 Wrocław, Poland; jerzy.witkowski@pwr.edu.pl

**Keywords:** soil moisture, soil conductivity, soil impedance measurement, soil electric permittivity, dual-frequency meter, high-frequency measurements, dual-frequency sensor

## Abstract

The measurement of soil water content is a very important factor in plant cultivation, both from an economic and ecological point of view. Proper estimation of moisture content not only allows for proper yields but can also contribute to ecologically appropriate use of fresh water, of which the world’s resources are limited. It is important, for example, that the moisture content in the root area of plants is optimal for their growth, while over-watering can result in losses in the form of water, which seeps below the root layer and is lost. The novel, inexpensive electronic meter for measuring soil moisture is presented in the article. The meter, based on a capacitive method, uses an optimization algorithm to calculate soil electrical permeability and a simplified new formula between soil electrical permeability and volumetric moisture content. Moreover, by using two high-frequency signals for measurements, it is possible not only to estimate moisture content but also soil conductivity. Both readings obtained from the meter not only allow for rational management of crop optimization for economic reasons but are also important for environmental protection. In addition, the inexpensive meter, based on the principle of operation presented, can be made as an IoT module, which allows for its wide application.

## 1. Introduction

Water brings life and is indispensable for life. This observation dates back to antiquity. More specifically, water allows for vegetation to grow, but its amount should remain within certain limits. Both too much water and too little water are harmful. The advances in agricultural science make one dream of not only qualitative but also quantitative analysis of the relationship between economically optimal plant growth and the moisture content of the soil. Consequently, a number of concepts related to such estimation have been developed. The earliest source authors stumbled upon comes from 1889 citated in [1]. Already, this very early source mentions these notions, which proves that the beginnings of the scientific approach to these questions are even earlier. Another paper [1] makes full use of these notions and shows scientific approach in their application.

The basic notions related to soil moisture content and its impact on plant vegetation include the following [2]:

- Field capacity (FC) [1,3];
- Water potential (WP) [4,5];
- Permanent wilting point (PWP) or wilting point (WP) [3];
- Available water capacity (or available water content (AWC))—field capacity of water available for vegetation;
- Water content (or moisture content)—percentage share of water in soil calculated as a percentage of volume (volumetric moisture) or percentage of weight (gravimetric moisture).

Volumetric moisture content can be calculated as pure water content per dry volume or per total volume before drying, which is often further limited by the condition of not disturbing the sample (sample category A according to EN22475-1). Similarly, gravimetric moisture content is calculated as the ratio of the mass of water in the soil to the mass of the dry soil sample or (more often) to the mass of the soil sample before drying.

**Figure 1:**
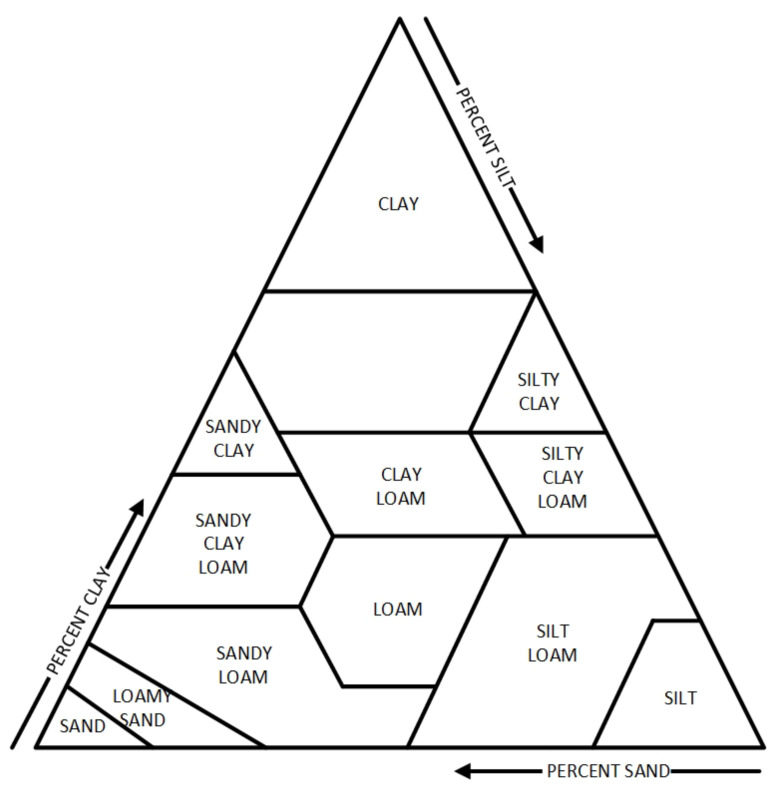
Soil textural triangle. Source: United State Department of Agriculture.

Most of the listed concepts (except water content), although important for plant breeding, do not lend themselves to quantitative analysis or are difficult to measure (such as potential). This is due to the huge variety of soil types (Figure 1) and their ambiguous definitions. Therefore, to determine the optimal amount of water in the soil, retention charts or water holding capacity charts [4] are introduced.

**Figure 2:**
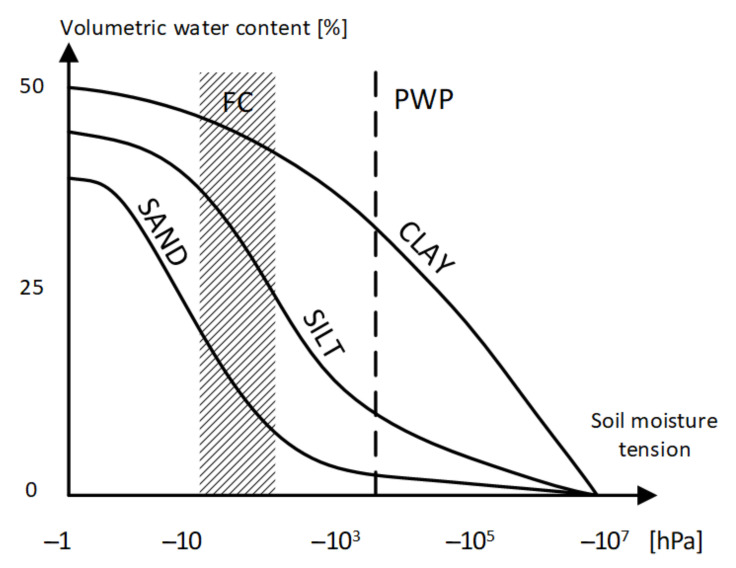
Illustrative retention curves for basic soil types (adapted from [4]).

**Figure 3:**
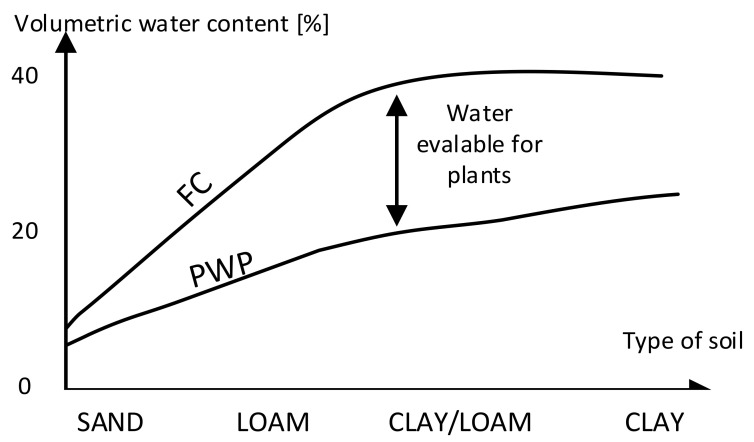
Typical curves of water capacity (FC) and permanent wilting point (PWP) vs. soil type (adapted from [6]).

An example of a water retention curve, demonstrating the relationship between volumetric moisture and field potential, is shown in Figure 2 [4], and an example of water holding capacity charts is shown in Figure 3. These curves are empirical. Their practical use is difficult in that they must be drawn for each type of soil separately, and the number of soil types, as said, is huge and is difficult to strictly define (Figure 1) [5].

Therefore, a practical approach to the question of determining the optimum soil moisture can be reduced to the measurement of quantities that are easy to measure using simple physical methods. Then, experimental data (empirical curves like in Figure 3) can be used in order to determine the irrigation of soil required for correct cultivation.

Although soil water content (volumetric or weight) is strictly defined, the measurement of these quantities is labor-intensive, requires a longer period of time and is not suitable for in situ measurements. For these reasons, a wide variety of methods have been developed for measuring physical quantities that can then be used to determine soil water content:

Tensiometric method—This method determines the soil moisture tension or water potential transmission [1,3,7].
Pressure plates method—In principle, this method gives the available water capacity [8,9,10,11]; however, it is labor-intensive and not suitable for in situ measurements.
Direct or indirect conductance measurement method [12,13,14]—This method measures the electrical conductance of water content. It is simple and inexpensive. However, one must remember that the measurement of conductivity strongly depends on the ion content (salinity) of the soil.
The TDR method (time domain reflectometry)—[15,16,17,18] By measuring high-frequency electromagnetic wave propagation along a longline in the soil, one can determine the permittivity of the soil and then the amount of water. The high-frequency apparatus needed for this method is expensive.
The FDR method (frequency domain reflectometry)—[19,20,21] This method is similar to the previous method with the difference that wave analysis is performed in the frequency domain, not the time domain. It is also expensive.
The DPHP method (dual-probe heat pulse)—[8,12,22]—This method measures the thermal conductivity, which is a function of water content. The construction of the meter is not very expensive, but a meter of this type is energy-intensive, so the operating time on battery power, especially for in situ measurements, is short.
The GPR method (ground penetration radar)—[12] This method analyzes the reflection of high-frequency electromagnetic waves from layers of the ground. The method is applicable when searching for large underground objects or when performing geological searches. In agriculture, it is not applicable.
Isotope method—[2,23,24] This is the most useful method and is based on neutron scattering. The highest neutron scattering occurs for the atoms with the lowest atomic mass, namely hydrogen. Therefore, measuring neutron scattering provides information about water content.
Optical method—This method is based on the difference in absorption of optical radiation (often infrared) waves of different wavelengths or the change in polarization of the reflected light wave. It is used to search large areas, for example, from an aircraft or even from a satellite [2,8].

The solutions listed above have the following drawbacks and inconveniences:

- The gravimetric method—This method is very laborious and impossible to perform in situ.
- The tensiometric method (with a manometer replaced by an electronic MEMS sensor)—Its good point is that it measures a physical value that is close to field capacity. However, periodic maintenance may be required; the porous ending may need replacement and the response time is rather long (up to several hours). The measurement is sensitive to improper placement of the system in soil.
- The pressure plates method—In principle, this method does not measure moisture but field capacity and is not an in situ method.
- The conductance measurement method with plaster blocks—This method requires frequent maintenance and replacement of blocks. The absolute measurement has a high error due to the varying content of salt (fertilizers) and strongly depends on the contact between the electrode and soil.
- The conductance measurement method using metal probes—This method has low repeatability and is good for comparative measurements, i.e., when mapping terrain.
- TDR—This is a fairly accurate method, but equipment is expensive, as the sampling frequency required is high—in the range of 1 GHz. Without specially dug pits, measurement at different depths is not possible.
- FDR—Similar to TDR, this method is costly, as frequency sweeping is required; moreover, an expensive DDS generator must be used.
- Probes with heaters—They consume a lot of energy; battery power is not practicable.
- The isotope method—This method is very accurate but expensive; it requires training in the operation of radioisotope sources.

Capacitive method (mentioned and compared to other methods in [14,15,16,17,18,19,20,21,22,23])—This method measures the electrical capacity of a capacitor with soil as the dielectric. The dielectric permittivity of rock (mainly in the form of sand) ranges from 2 to 6 and that of air is 1, whereas the value for water is 80 (at 20 °C). Such a huge difference between water and all other components allows one to measure the moisture by determining the relative permittivity of soil, and based on that, water content can be calculated. In fact, the soil may contain impurities in the form of materials with relatively high magnetic permeability (some types of clay—[19]), but their content is low and they are so rare that they are practically insignificant.

This method is used by the authors and described in the next part of the paper.

## 2. Construction of the Sensor

From the point of view of application, the capacitive probe is simple and inexpensive. Its advantages include the following:

- Low energy consumption;
- Low cost of development;
- No need for periodic maintenance.

A possible drawback of the capacitive method is its lower accuracy compared to the TDR or neutron method [8,12,23,25], but one must also keep in mind its sufficient repeatability [8,12,25] and the relatively well-described methods of calibration [26,27,28,29].

**Figure 4:**
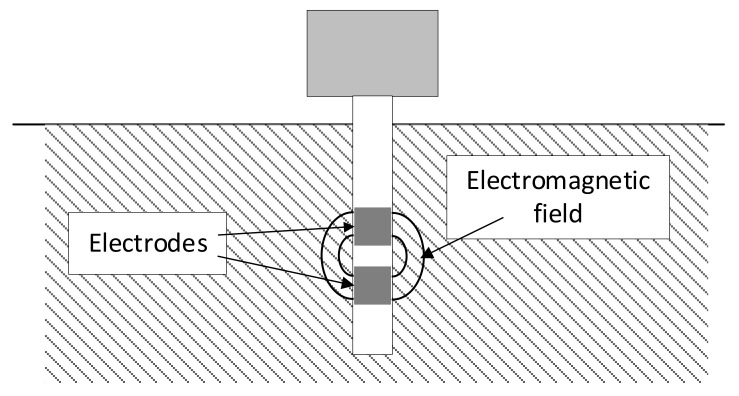
Basic illustration of capacitive measurement principle.

In [19,23], measurements using various probes are compared. A general schematic of capacitive measurement is shown in Figure 4, although probes similar to those from the TDR method are also in use [23]. Similar to TDR and FDR, the key to carrying out measurements using this method lies in the dependence of the dielectric permittivity of soil on the amount of water.

### 2.1. Sensor Design—Principle of Operation

**Figure 5:**
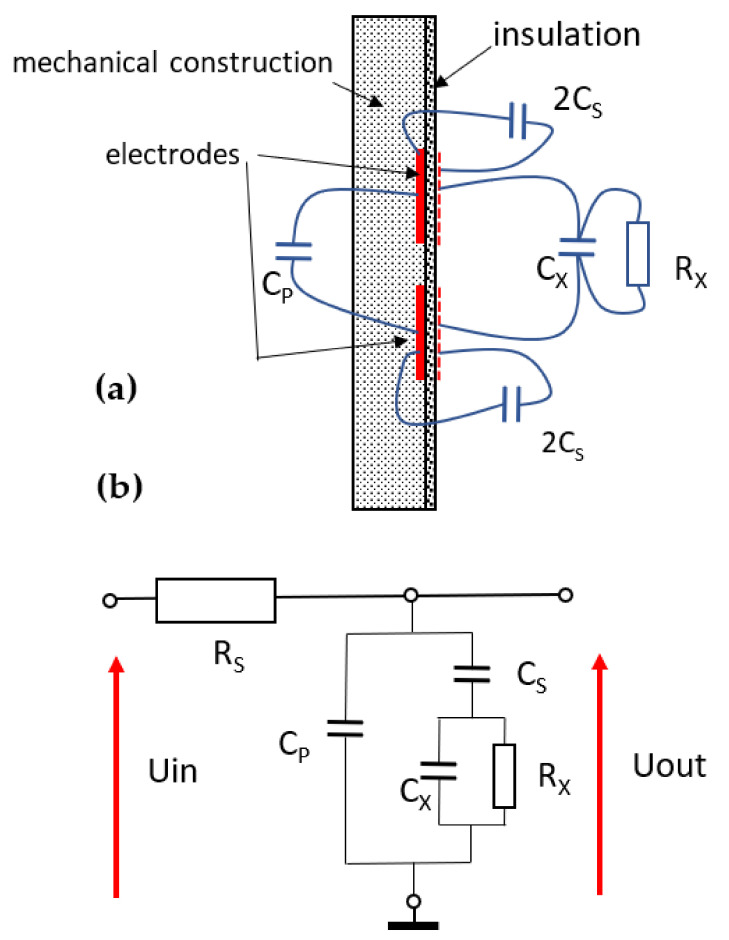
Measuring electrodes (**a**) and their equivalent circuit (**b**). (Rs—resistance in series to the probe; Cs—the capacity of the electrodes to the medium; Cp—the self-capacity part of the capacitor due to its construction; Cx—capacity being measured; Rx—the loss of the dielectric being measured).

The model constructed by the authors consists of two electrodes inside the dielectric pipe with a diameter of 30 mm, a wall thickness of 2 mm and an electronic circuit inside. An equivalent electrical diagram of the probe is seen in Figure 5.

**Figure 6:**
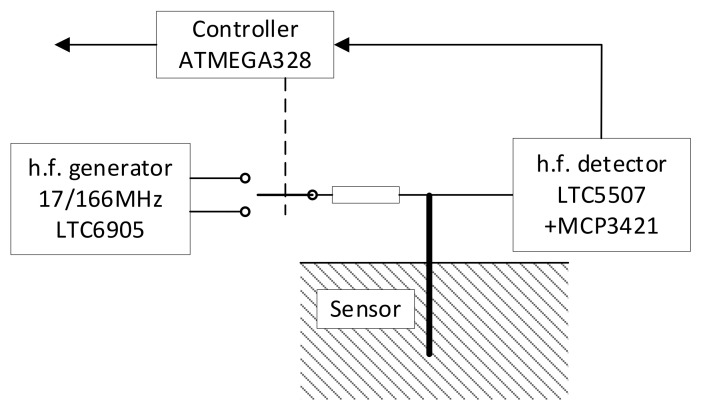
Basic illustration of capacitive measurement principle. The types of major integrated circuits used in the sensor prototype are given.

The construction diagram and the equivalent circuit are shown in Figure 6. *Cp* is the self-capacity part of the capacitor due to its construction, which is independent of the dielectric being measured, whereas *Cx* is the part of this capacitor whose dielectric measures the substance. *Cs* is the capacity of the electrodes to the medium being measured. The resistance Rx represents the loss of the dielectric being measured.

The measurement algorithm boils down to the determination of *Cx* and *Rx* based on the measurement of the impedance of the equivalent circuit for the values of *Rs*, *Cs*, *Cp* and *Cx*0 = *Cx*(*ε_w_* = 1) determined during calibration.

As seen in Figure 6, the equivalent circuit transmittance can be expressed as follows:

(1) $$T\left(f;Rs,Cp,Cs,Cx0;\varepsilon_{w},\sigma \right)=\left|\frac{U_{out}}{U_{in}}\right|=\;=\left|\frac{1}{1+R_{s}\left(j\omega C_{P}+\frac{1}{\frac{1}{j\omega C_{S}}+\frac{1}{j\omega C_{X}+\frac{1}{R_{X}}}}\right)}\right|,$$

where *ε_w_* and *σ* are the soil relative permittivity and conductivity, respectively, *ε_0_* is the vacuum permittivity and

(2) ω=2πf,

(3) $$C_{X}=\varepsilon_{w}C_{X0},$$

(4) $$R_{X}=\frac{\varepsilon_{0}}{{\sigma C}_{X0}}.$$

Calibration consisted of minimalizing the expression:

(5) $$\sum_{i=1,2;\;\varepsilon_{w}=nown}\left\|\;\;T_{measured}\left(f_{i};Rs,Cp,Cs,Cx0;\varepsilon_{w},0\right)-T\left(f_{i};Rs,Cp,Cs,Cx0;\varepsilon_{w},0\right)\right\|.$$

That is, the difference between the model’s transmittance function and the measured transmittance function for two frequencies *f*_1_ = 17 MHz and *f*_2_ = 166 MHz and several nonconductive media of known permeability is minimized. As a result, we obtain the model parameters: *Rs*, *Cp*, *Cs* and *Cx*0.

Calibration is time-consuming, but it can be performed once using a PC and a program such as Matlab or Octave.

Finally, using the model parameters *Rs*, *Cp, Cs* and *Cx0*, the function

(6) $$\sum_{i=1,2;\varepsilon_{w},\sigma =unnown}\left\|\;\;T_{measured}\left(f_{i};Rs,Cp,Cs,Cx0;\varepsilon_{w},\sigma \right)-T\left(f_{i};Rs,Cp,Cs,Cx0;\varepsilon_{w},\sigma \right)\right\|$$

is minimized with respect to *ε_w_* and *σ* for two frequencies, *f_i_*, equal to 17 MHz and 166 MHz.

The sensor (Figure 6) consists of a high-frequency circuit for the measurement of dielectric permittivity. This circuit includes a tunable generator and a h.f. detector. The generator is controlled by a processor using digital signals, which allows it to achieve an output frequency of 17 MHz or 166 MHz. The detector voltage is processed in a delta-sigma A/D converter and transferred to the controller.

### 2.2. Example of Calibration and Measurement Algorithm

The following were used for calibration [27]:

- Air—*ε_w_* = 1;
- Glass balls with a diameter of 0.4–0.6 mm—*ε_w_* = 3;
- Isopropanol (propan-2-ol, isopropyl alcohol)—*ε_w_* = 20 (25 °C);
- Water solution with isopropanol—*ε_w_* = 32;
- Water solution with isopropanol—*ε_w_* = 40;
- Distilled water (*σ* < 5 µS/cm)—*ε_w_* = 80.

The following measurement results were obtained.

In order to develop a model, the following realistic ranges of model variables were assumed:

*Rs* = 120–135 Ω, *Cp* = 6.5–12 pF, *Cs* = 10–20 pF, *Cx*0 = 0.5–1 pF and *Rx* = 10 MΩ, σ = 0.

Applying Equation (5) to the data in Table 1, a model of the sensor has been obtained with the following parameters: *Rs* = 0.125 kΩ, *Cp* = 10.6 pF, *Cs* = 13.5 pF and *Cx*0 = 0.7 pF. As seen, the parameters of the model we obtained are within the assumed ranges.

### 2.3. Measurement Example

As an example of an unknown medium, a mixture of isopropanol, distilled water and tap water was tested. Detector ridings were normalized to those obtained for *e_w_* = 1 and are listed in Table 2.

The measurement consists of reading the voltage from the detector for the unknown medium for frequencies 17 MHZ and 166 MHz and finding the minimum of Equation (6) with respect to *σ* and *ε_w_*. For these data, the final measurement results are *ε_w_* = 32 and *σ* = 0.49 dS/m, which are consistent with expectations and coincide with the parameters of the medium being measured. Validation was carried out by the measurement of parameters using an RLC bridge, the standard air capacitor located in the investigated medium (the measurement of epsilon) and an electrical conductivity meter.

**Figure 7:**
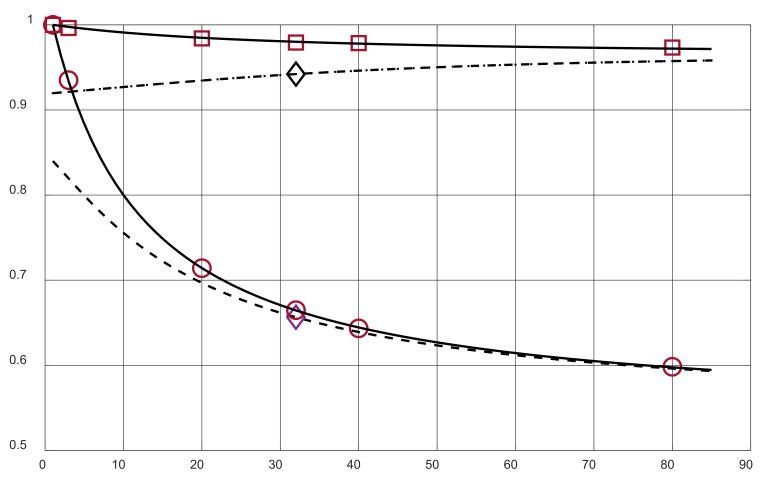
Calibration curves and their measurement points for 17 MHz (solid line and □) and for 166 MHz (solid line and O); measurement result point (*ε_w_* = 32, *σ* = 0.49 dS/m) against transmittance curves (dashed lines and “rhombus”) for *f*_1_ = 17 MHz (top line) and *f*_2_ = 166 MHz (bottom line).

The calibration and measurement results are illustrated in Figure 7, where the measurement points made for calibration, as well as the final measurement point, can be seen against the transmittance curves. It is worth mentioning that the optimization procedures for both the calibration and the final result, in terms of *σ* and *ε_w_*, were carried out using the “least squares” procedures.

**Figure 8:**
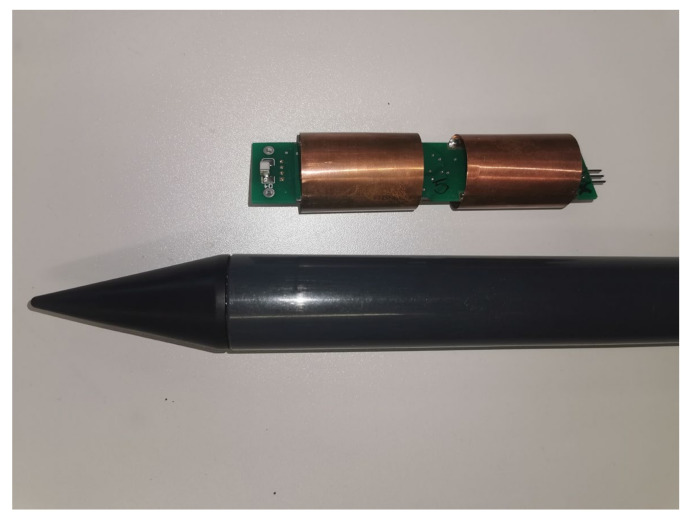
Prototype of the sensor and its housing.

The prototype constructed by the authors consists of two electrodes inside a dielectric pipe with a diameter of 30 mm, a wall thickness 2 mm and an electronic circuit inside. Figure 8 shows the prototype of the sensor and its housing.

### 2.4. Electric Permittivity of Soil vs. Volumetric Moisture

As electric permittivity is the result of the algorithm described above, the moisture can be calculated. This means the relation $\theta =f\left(\varepsilon_{w}\right)$ is needed.

The relative permittivity of soil is not linearly dependent on the soil components. The interactions between water and solid particles must also be accounted for. It turns out that the relative permittivity of water in the form of a thin film covering a solid is around 3.6 rather than 80. Many other mutual relationships between water, rocks and air also need to be taken into account [24].

In the literature, the empirical equation of [16] is the most frequently quoted:

(7) $$\varepsilon_{w}=3.03+9.3\cdot \theta +146\cdot \theta^{2}-76.7\cdot \theta^{3}.$$

Another relationship can be found in [29]:

(8) $$\varepsilon_{w}=2.87-11.1\cdot \theta +276\cdot \theta^{2}-272\cdot \theta^{3}.$$

However, the same lead author in [17] and another one in [19,27] suggest a more complex relationship:

(9) $$\varepsilon_{w}^{\alpha }=\varepsilon_{water}^{\alpha }\cdot \theta_{water}+\varepsilon_{air}^{\alpha }\cdot \theta_{air}+\left(1-\emptyset \right)\cdot \varepsilon_{minerals}^{\alpha }+\varepsilon_{ice}^{\alpha }\cdot \theta_{ice},$$

where *α*—a coefficient that depends on the distribution of particles, which is 1/2 of the normal distribution, *θ*—volumetric content of water, ice and air, respectively, *ε*—relative permittivity of water, ice and air, as in the subscript, *φ*—porosity.

The following sophisticated formula comes from [30]:

(10) $$\varepsilon_{w}=2.37+\left(-5.24+0.55\times \left(\%sand\right)+0.15\times \left(\%clay\right)\right)\theta +\left(146.04-0.74\times \left(\%sand\right)-0.85\times \left(\%clay\right)\right)\theta^{2}.$$

Yet another relationship [27] (for *ε*_rock_ = 4, *ε*_water_ = 80) is described as follows:

(11) $$\varepsilon_{w}=a_{0}\left(\emptyset \right)+a_{1}\left(\emptyset \right)\cdot \theta +a_{2}\left(\emptyset \right)\cdot \theta^{2}+a_{3}\left(\emptyset \right)\cdot \theta^{3},$$

where

(12) $$\begin{array}{c} a_{0}\left(\emptyset \right)=2.35\times \emptyset^{-0.398},\\ a_{1}\left(\emptyset \right)=-49.54+509.7\times \emptyset -1241.5\times \emptyset^{2}+839.7\times \emptyset^{3},\\ \begin{array}{c} a_{2}\left(\emptyset \right)=513-3708.9\times \emptyset +9129.8\times \emptyset^{2}-6562\times \emptyset^{3},\\ a_{3}\left(\emptyset \right)=-487.5+5605\times \emptyset +14.717\times \emptyset^{2}-11.115\times \emptyset^{3}, \end{array} \end{array}$$

and Ø is the porosity of the soil.

In [31], a comparison is given between the above approaches and the authors’ own method, which requires several other parameters, such as “the active surface of solid components”.

Another relationship has been obtained by the authors of [20,26] (now permittivity to volumetric moisture):

(13) $$\theta =0.0838\sqrt{\varepsilon_{w}}-0.0846.$$

Very common equations can be found in [26,28,31]. In general the formula can be written as follows:

(14) $$\theta =A\sqrt{\varepsilon_{w}}+B;$$

however, the coefficients A and B are different and depend on the type of soil. The value of A changes in the range from 0.07 to 0.18, and B changes from −0.039 to −0.29.

As seen, there are a lot of comprehensive approaches to the problem. Many of them are very sophisticated. Comparison between them can also be found in [32]. Above are quoted only a few formulas that can be found in the literature that determine the relationship between relative permeability and volumetric moisture content. Some of them are really complicated and use many parameters, so they are difficult to use in practice.

The authors propose a new, much simpler model, expressed as follows:

(15) $$\theta =A{\left(\varepsilon_{w}-\varepsilon_{wDS}\right)}^{0.6},$$

where *ε_wDS_* is the dielectric permittivity of dry soil (rock) and *A* is an arbitrary coefficient.

**Figure 9:**
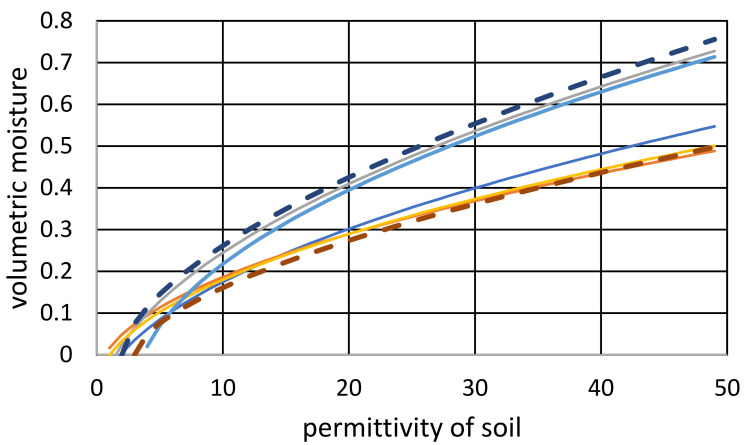
Volumetric moisture as a function of dielectric permittivity. Dashed lines are obtained from Formula (15) for *(A*; *ε_w_*) = (0.075; 2) and (0.05; 3), respectively. Continuous lines are plotted based on the experimental data from [16,24].

Such a formula only requires the selection of an appropriate parameter *A*, but the second parameter *ε_wDS_* is easy to interpret and guess. The correctness of such an approach is shown in Figure 9, where the relationship (15) is plotted for two values of *A* and *ε_wDS_* on the background of curves taken from the experimental data presented in [16,24]. As seen, the accuracy can be considered satisfactory or better than satisfactory, especially given the discussion on accuracy presented in the next section of the paper.

## 3. Accuracy Discussion

**Figure 10:**
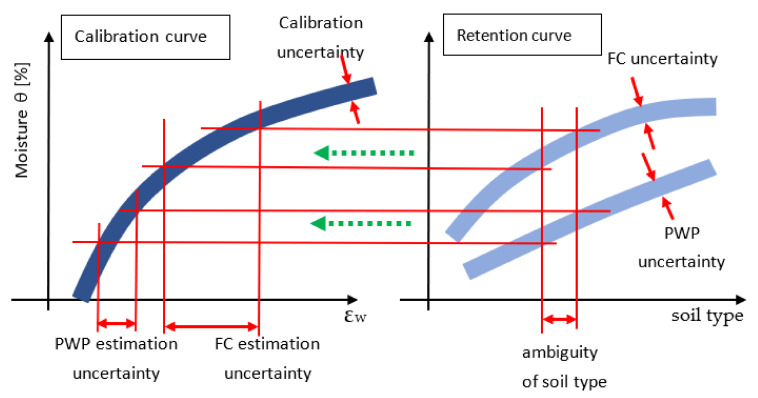
Overall uncertainty of irrigation control.

Figure 10 shows the construction of irrigation control uncertainty based on the measurement of the dielectric permittivity of the soil. The example seems to confirm that the main problem is not the measurement of permittivity, as such, but the uncertainties that add up to it, related to the calibration *θ* = *θ*(*ε_w_*), the PWP curves, the FC curve and the soil type.

In conclusion, Approximation (15) should be used in the meter, but the coefficients should be selected according to the type of soil. In addition, it is clear that trying to find the exact relationship $\theta =f\left(\varepsilon_{w}\right)$ is not necessary due to the relatively large uncertainty in determining the soil type and PWT and FC curves (Figure 10).

### Temperature Adjustment

In [31], a formula for temperature adjustment of the dielectric constant of water is given. In the temperature range of 0–30 °C, the linear part of this formula (a higher rank approximation is practically neglected) is

(16) $$\varepsilon_{H02}=78.5\left(1-0.00458\left(t{}^{\circ}[C]-25\;{}^{\circ}C\right)\right)$$

where *t* is the temperature in Celsius.

It follows that the permeability of the soil will also change with the temperature. Such changes also apply to conductivity. Practical relationships can be found in [33,34,35,36,37], among others. They can be reduced to the following relationship:

*ε*_25_ = *ε_m_* − 0.114(*t*[°C] – 25 °C), (17)

where *ε*_25_ is the permeability at 25 °C, and *ε_m_* is the measured value at *t*[°C].

Similarly, for conductance,

(18) $$\begin{array}{c} \sigma_{25}=\sigma_{m}(1-0.02035\left(t\left[{}^{\circ}C\right]-25\;{}^{\circ}C\right)+0.3822\times 10^{-3}{\left(t\left[{}^{\circ}C\right]-25\;{}^{\circ}C\right)}^{2}-\\ \;\;0.555\times 10^{-6}{\left(t\left[{}^{\circ}C\right]-25\;{}^{\circ}C\right)}^{3}). \end{array}\;\;\;$$

In this case, the relationship given is an approximation by as much as the third order because the relative changes in conductance are greater than the changes in permittivity. However, it seems that for practical applications, linear dependencies are sufficient.

One approach to control temperature-related changes can only affect the result by altering the parameters of the measured quantities (humidity and conductivity) and the sensor reading due to changes in the electronics using a factor of the above equations. Of course, the meter should be additionally equipped with a soil temperature sensor.

Another approach is to ignore thermal changes in permeability from temperature as they can cause inaccuracies due to a mismatch between calibration and soil type (Figure 10). In the case of conductivity, the thermal changes are greater, but usually, the accuracy of the measurement is lower, and user expectations in this regard are usually not high because it is often sufficient to observe the changes in the parameter readings rather than their absolute value.

## 4. Summary

The dual-frequency measuring probe presented in this work allows for simultaneous estimation of soil moisture and conductivity within the ranges of *ε_w_* = 1–80 and *σ* = 0–5 ds/m. Before the first measurement, the system requires calibration by measurements in several nonconducting standard media—air (*ε_w_* = 1), distilled water (*ε_w_* = 80) and, e.g., isopropanol (*ε_w_* = 20) and some mixtures of the two last media. As a result, the calibration parameters Rs, Cp, Cs and Cx0 for the investigated measurement probe were determined.

Calibration involves taking a complex probe model and recognizing its parameters based on optimizing the differences between the absolute value of the transmittance function and its measurements for two frequencies and several standard solutions.

After the calibration procedure, the probe measures its transmittance together with the measured medium, which allows for the conductivity and water content of the medium to be estimated. Both algorithms for calibration and determination of *ε_w_* and *σ* optimization procedures are used.

Similarly, the estimation of the measurement results involves minimizing the difference between the absolute value of the transmittance function with the model parameters assumed during calibration and the measured values for two of the same frequencies, as in the case of calibration.

Moreover, the moisture of the soil is calculated using a new relationship, (15), proposed by the authors. This relationship has been compared with many measurements taken from the literature. Its relationship is much simpler. Only two values are needed to determine the water content of the soil, with one of them (*ε_wDR_*) having a simple physical interpretation.

## Figures and Tables

**Table 1:** Detector signals for several nonconductive mediums normalized to signal for *ε_w_* = 1 (air).

*f*/*ε_w_*	1	3	20	32	40	80
17 MHz	1	0.9963	0.9841	0.9792	0.9785	0.9734
166 MHz	1	0.9350	0.7141	0.6647	0.6433	0.5982

**Table 2:** Detector signals for unknown medium (mixture of isopropanol, distilled water and top water) normalized to signal for *ε_w_* = 1.

*f*/*ε_w_*	ε_w_ = 1	ε_w_ = ??
17 MHz	1	0.9422
166 MHz	1	0.6564

## Data Availability

The data presented in this study and the Matlab code of the discussed algorithms are available from https://github.com/AGwroc/moisture_sensor (accessed on 30 April 2024).
